# A qualitative exploration of care-seeking pathways for sick children in the rural Oromia region of Ethiopia

**DOI:** 10.1186/s12913-017-2123-5

**Published:** 2017-03-09

**Authors:** Bryan Shaw, Agbessi Amouzou, Nathan P. Miller, Jennifer Bryce, Pamela J. Surkan

**Affiliations:** 1 Department of International Health, Institute for International Programs, Johns Hopkins Bloomberg School of Public Health, Baltimore, USA; 2 Department of International Health, Johns Hopkins Bloomberg School of Public Health, Baltimore, USA

**Keywords:** Careseeking, Child health, Health care utilization, Rural communities, Ethiopia, Qualitative research

## Abstract

**Background:**

Ethiopia has experienced rapid improvements in its healthcare infrastructure, such as through the recent scale up of integrated community case management (iCCM) delivered by community-based health extension workers (HEWs) targeting children under the age of five. Despite notable improvements in child outcomes, the use of HEWs delivering iCCM remains very low. The aim of our study was to explain this phenomenon by examining care-seeking practices and treatment for sick children in two rural districts in the Oromia Region of Ethiopia.

**Methods:**

Using qualitative methods, we explored perceptions of child illness, influences on decision-making processes occurring over the course of a child’s illness and caregiver perceptions of available community-based sources of child illness care. Sixteen focus group discussions (FGDs) and 40 in-depth interviews (IDIs) were held with mothers of children under age five. For additional perspective, 16 IDIs were conducted fathers and 22 IDIs with health extension workers and community health volunteers.

**Results:**

Caregivers often described the act of care-seeking for a sick child as a time of considerable uncertainty. In particular, mothers of sick children described the cultural, social and community-based resources available to minimize this uncertainty as well as constraints and strategies for accessing these resources in order to receive treatment for a sick child. The level of trust and familiarity were the most common dynamics noted as influencing care-seeking strategies; trust in biomedical and government providers was often low.

**Conclusions:**

Overall, our research highlights the multiple and dynamic influences on care-seeking for sick children in rural Ethiopia. An understanding of these influences is critical for the success of existing and future health interventions and continued improvement of child health in Ethiopia.

**Electronic supplementary material:**

The online version of this article (doi:10.1186/s12913-017-2123-5) contains supplementary material, which is available to authorized users.

## Background

Ethiopia has recorded notable progress in child health outcomes, including meeting the fourth Millennium Development Goal (MDG-4) target of reducing under-five child mortality by two-thirds since 1990 [1]. Much of this success has been attributed to the rapid expansion of health care infrastructure, primary care coverage and scale up of community-based child health interventions to Ethiopia’s underserved and largely rural communities [2, 3]. Despite these improvements, utilization of biomedical sources of care for sick children remain low, with less than one-third of caregivers seeking care from these sources for a child sick with diarrhea, malaria or pneumonia. Consequently, rates of use of evidence-based child illness treatments such as oral rehydration solution (ORS) for diarrhea, antimalarials for malaria and antibiotics for pneumonia are also low [4].

To try to account for these trends, most public health studies are quantitative and focus on specific barriers to access such as cost and transportation or key demographic characteristics associated with use of biomedical providers [5, 6]. However, these models have been criticized as reductive and detached from the everyday decisions of health care consumers [7, 8]. They also miss out on health behaviors and actions that take place outside the formal care sector [9]. Concerns such as cost and distance are limited in accounting for care-seeking choices [10]. These studies often investigate the pathways at specific points (e.g., recognition of illness, decision to seek care, choice of care provider, etc.) over the course of a child illness episode [8, 11]. Each of these steps is influenced by a range of sociocultural norms, local decision-making practices and socioeconomic constraints interacting within local and national healthcare systems [12, 13]. In the context of local sociocultural realities, care-seeking actions are often pragmatic and based on distinctions such as familiarity with a source as opposed to those between “appropriate/inappropriate” or “biomedical/traditional” care [14]. Thus, focusing solely on the use of biomedical options limits the ability to understand the full range of care-seeking behaviors.

Although low rates of use of formal biomedical treatments have been widely documented in rural Ethiopia [15, 16], few studies have qualitatively explored reasons for this phenomenon. Most service utilization studies in Ethiopia occurred prior to significant investments in national primary and child health programs such as the scale up of the health extension program (HEP) and inclusion of community case management for common childhood illnesses delivered by health extension workers (HEWs) at rural health posts. Furthermore, none have examined the sociocultural context of care-seeking for common childhood illnesses or elicited care-seeking narratives from caregivers themselves. Consequently, little is known about caregiver practices for seeking care and treatment for sick children throughout the course of a child’s illness. Finally, recent large-scale, quantitative surveys conducted in rural Ethiopia have suggested extremely low rates of care-seeking from informal sources such as traditional healers and unlicensed drug vendors [17].

This study aims to explore when, where and why caregivers seek care and treatment when they believe their child is ill. It seeks to provide context for findings of low utilization of formal biomedical sources where evidence-based child illness treatments can be obtained in rural Oromia, Ethiopia. These sources include HEWs located at health posts within rural communities as well as health centers and private clinics in nearby (approximately 5–30 km) towns.

## Methods

Qualitative research was conducted to aid in the evaluation of Ethiopia’s scale-up of integrated community case management (iCCM) of common childhood illnesses in rural Oromia region. Data collection was conducted from December 2012 to January 2013. Eight rural *kebele* sites, each corresponding to one health post catchment area serving approximately 5000 individuals, were purposively selected from sites where iCCM implementation and scale up has been occurring for at least 2 years in the Jimma and West Hararghe zones. Purposive selection was based on existing information for health post utilization and distance from nearest health referral facility located in an urban center. Sites were selected to achieve maximum variation for these factors. Characteristics of selected *kebele* sites are presented in Table 1.

**Table 1:** Characteristics of selected *kebele* sites

	Woreda	Utilization^a^	Distance to referral facility (in km)	Catchment size (in square km)	Number of functional HEWs
Jimma					
Site 1	Goma	Low	23	16	1
Site 2	Omo Nada	Medium	8	9	2
Site 3	Shebe Seneba	Medium	31	20	2
Site 4	Kersa	High	10	8	3
West Hararghe					
Site 5	Oda Bultum	Low	5	10	1
Site 6	Gemechis	Medium	3	17	2
Site 7	Boke	Medium	25	42	1
Site 8	Boke	High	13	21	1

Source: Miller N. *Integrated Community Case Management of Childhood Illness in Ethiopia: Implementation Strength and Quality of Care*. Dissertation Report. Baltimore: Johns Hopkins University; 2013

^a^Low: 0–10 sick child consultations/month; medium: 10–40; high: >40

The study design was informed by rapid ethnographic assessments developed as part of applied anthropological research methods focusing specifically on child health and care-seeking behaviors [18, 19]. One team of four (2 males, 2 females) college-educated, Afan Oromo-speaking investigators with experience in qualitative research methods were trained and conducted the research. Qualitative methods consisted of focus group discussions (FGDs) and in-depth interviews (IDIs) conducted in the Afan Oromo language. FGDs focused on social norms of care-seeking and community perceptions of local sources of care. IDIs focused on eliciting a narrative of care-seeking experiences and strategies during the most recent child illness [20].

Upon entering a selected *kebele*, the research team met with local leaders and health post staff to explain the goals and conduct of the research. Study participants were purposively selected with the assistance of community leaders, voluntary community health workers (VCHWs) and local members of the Health Development Army. Investigators also canvassed *kebele* villages and invited those present to increase representation from all sub-villages. Sampling for FGDs focused on mothers of children under the age of five. Sixteen FGDs were held, with eight each in Jimma and West Hararghe. Forty IDIs were held with mothers of children under the age of five screened for having experienced a recent child illness over the previous 1 month. For additional context and perspectives, 16 IDIs were held with a subset of these women’s husbands and 22 IDIs were held with HEWs (mostly in the Amharic language) and VCHWs. Table 2 gives the sample size for each method and population. All research interactions were conducted in Afan Oromo and with verbal consent and no informants refused participation. Oral rather than written consent was obtained due to the large number of non-literate informants and potentially sensitive nature of questions about government child health programs. FGDs took place in geographically convenient locations such as schools or community meeting places while IDIs took place in informants’ homes and only participants were included in interviews. FGDs and IDIs ranged from 30 to 90 min. FGD and IDI study instruments are provided in Additional file 1. The study received ethical approval from the Oromia Region Health Bureau and Johns Hopkins Bloomberg School of Public Health.

**Table 2:** Sample size, by method

Technique, Population	Per site	Total (for all sites)
FGD, Mothers	2 (6–12 individuals)	16 (132 individuals)
IDI, Mothers	4–6	40
IDI, Fathers	2	16
IDI, HEWs	1–2	10
IDI, Volunteer/HDA	1–2	12
*Total*	*10–14*	*94*

FGDs and IDIs were audio-recorded and transcribed into English and notes were taken during and after research interactions. Back translation of selected areas of text was performed to assess for quality of translation. Qualitive data were analyzed using Atlas.ti software (Development SS, Berlin). Charmaz’s grounded theory perspective was used to guide data analysis [21]. Dominant themes were identified through open coding of a select number of transcripts to construct a code book. Axial coding, relating open codes to each other, was also performed. Memos were created to organize patterns in key themes emerging from the data and visual representations (e.g., patterns of sources used). All coding was performed on each transcript by the primary author and two graduate student assistants. Codes were compared and reconciled to create a final codebook, from which the transcripts were coded. During analysis, data were compared across sites, methods and participant groups to triangulate findings.

## Results

### Conceptualization of child illness

For many participants in this study, child illness was seen as a deviation from an ideal state of *joli si’a ina qaba* (“child full of health/energy”). This was often contrasted with *joli lafu*, a term denoting both acute and long-term child “weakness.” *Lafu* was the most common initial symptom noted, recognized primarily by diminished activity, appetite and general lethargy. According to several FGD participants, traditional medicines (*qoricha Oromo* or *qoricha wabiya*), consisting of herbs and amulets given by the hand of a respected elder, are the correct treatment of *lafu*.

Participants made several distinctions in child illnesses based on perceptions of etiology, severity and appropriate treatment modality. The terms *ibidda* (Jimma) and *cimma* (West Hararghe) often indicated severe illnesses and were frequently attributed to a compounding of causal factors and distinguished from common, everyday child illnesses (*dhukuba joli*). The most common distinction was between illnesses and symptoms that should be treated with *qoricha Oromo* and those treated with *tijaniya* (“modern”) or biomedical drugs. In general, herbal medicines and more specialized *qoricha Oromo* were seen as appropriate for common illnesses, or those with suspected supernatural causes, and *tijaniya* medicines were thought appropriate for severe illnesses. Despite most participants subscribing to these distinctions, there was frequent debate and ambiguity when classifying symptoms into these categories as seen in the following exchange:

*P*_*1*_ *: There are many kinds of diarrhea in our children. There is white diarrhea. This is very cimma and requires the hands of an elder knowing qoricha Oromo to undo the work of buda nyate [evil eye].*

*P*_*2*_ *: She does not talk sense. We do not hold these wabiya [traditional] beliefs any longer. Diarrhea is because a child has eaten polluted foods. It becomes cimma because a mother does not go for tijaniya medicines at the health post.*

*P*_*3*_ *: It is God’s will if a child gets diarrhea.*

*P*_*4*_ *: But we must not let our children die. We must know the name of the illness and get medicines to prevent their suffering.* (FGD, mothers, West Hararghe)

Ultimately, there was considerable variation and overlap in perceptions of etiology, symptoms and severity of illnesses making generalizations across sites difficult.

### Labeling of child illness

For most child illness episodes, the mother was the first to recognize illness symptoms in their children. Upon recognition, most mothers consulted their lay referral network, commonly elderly family members and neighbor women with children, to seek advice in order to “give a name to the illness.” This act of labeling an illness was regarded as an essential action in order to understand the nature of an illness and to justify and facilitate a course of care-seeking actions:

*R: I suspected she [child] was ill when she stayed in the house and did not want to leave. After one day, she was lafu and I asked my neighbor to assist in giving a name to the illness because I was worried about taking the right steps to treat her. We agreed it was qora [similar to “flu” in Jimma] and to treat her with herbs in the home, but this became ibidda. My neighbor said that this was michi somba [“lung/breathing illness”] and that I should take the child to town for treatment.*

*I: You said you were worried about taking the right steps?*

*R: I do not want to worry my husband if she does not require money for treatment. We did not know the illness. I must know before worrying my husband.* (IDI, mother, 18–24 years old, agriculture, Jimma)

For others, especially those suggesting easy access to care providers, naming the illness was bound up in the care-seeking process as caregivers enlisted multiple sources of care and relied on care providers to name the illness.

Caregivers were often explicitly aware of the social meanings that proceeded from naming the illness. In FGD discussions of etiology, participants often supported the statement that: *“[Child illness] happens due to a mother’s carelessness. It is a mother’s duty to protect her children from harm.”* This was especially common in illnesses perceived as caused by exposure to supernatural forces or environmental extremes.

Aware of the potential for blame, caregivers actively negotiated illness interpretations to avoid blame:

*My husband’s mother said my actions were the cause of my child’s breathing problems and that I should not have taken her be attacked by the wind. I did not believe her words and I did not want others to accept her thinking that I neglect my child. I took the child to the health post because [the HEW] would tell me the cause.* (IDI, mother, 25–29 years old, daily wage work, West Hararghe)

Caregivers were also aware of the potential economic consequences associated with naming an illness. Illness interpretations were dynamic, contested, and often shifted with the inclusion of illness interpretations from “lay” and “expert” consultation. Listening to one illness interpretation over another could be a potential coping strategy when confronting constraints on preferred care-seeking strategies.

Most HEWs and VCHWs suggested that problems relating to recognition stemmed from a lack of awareness and knowledge of child illnesses. Some even suggested that this lack of awareness was due to “carelessness” and “ignorance” of mothers. Others, however, recognized that mothers are often aware and motivated to take their child for treatments, but are unable to do so due to a range of barriers, a perception supported by one VCHW: “*We give them education and they know to take their child for care, but they are poor. What can they do?”*

### Decision to seek care outside the home

Despite a general perception among HEW informants that “*mothers in this community will not leave their home when their child is sick*,” care and treatment was sought outside the household in nearly three-fourths of the care-seeking episodes discussed in this study. The decision to seek care outside the home often began with a perceived failure of self-treatment in ameliorating a child’s symptoms. Alternatively, some mothers suggested that home-based actions were successful in aiding interpretation of an illness and the illness warranted stronger treatments. For most caregivers, decisions to seek care outside the home were influenced by decision-making dynamics within a caregiver’s lay referral network, especially involving their husbands, and the resulting illness label and perceptions of severity.

Twelve caregivers took actions only in the home and stated that their child’s illness and symptoms resolved without complications. In most of these narratives, caregivers suggested a clear illness label, perceptions of severity were generally low and caregivers stated they had knowledge of effective home-based actions from previous child health episodes. For these reasons, there was less involvement of fathers and other members of lay referral networks, with a mother often making decisions herself or with the support of a limited number of others.

For the remaining mothers, illness interpretations were often uncertain. Most of these mothers explicitly expressed a desire to seek care outside their home in an effort to resolve both uncertainty and their child’s illness. This desire was especially pronounced when a mother perceived her child’s illness as becoming more severe:

*P: Home remedies are only for guba [common fever]. There are no home remedies for ibidda guba [severe fever]. If a mother keeps a child with ibidda guba in the home, that child will die. All mothers should know this and act urgently.*

*I: Why would a mother stay in the home if her child has ibidda guba?*

*P: She may be ignorant of the seriousness. She may be kept there by her husband. (FGD, mothers, Jimma)*

Whereas men were commonly regarded as passive during illness recognition, diagnosis and home-based care, most mothers suggested that they took a more active role in the decision to seek care outside the household. This was especially common if there were costs associated with care-seeking, treatment required long absences of the mother from her domestic responsibilities or transport of the child and securing treatments required the physical assistance of the father. In these instances, many mothers agreed that “*it is our duty to gain permission from our husbands to leave the home for treatment*.”

For fathers, the decision to seek care outside the home often depended on perceptions of financial costs associated with care-seeking and perceptions of efficacy of available treatment sources. Most saw their role in care-seeking as ensuring the least expensive and most efficacious mode of treatment, as related by the following father:

*Women are emotional and they can make bad decisions. There are many sellers of useless herbs and drugs and they easily cheat women. She [mother] would spend all of our family’s money for one child without thinking. When she is thinking of the child’s health, I am thinking of the family’s health.* (IDI, father, 30–34 years old, agriculture, Jimma)

Although men were commonly perceived as barriers to care-seeking outside the home in FGDs, most IDI informants suggested that, though their husbands held the ultimate authority in this decision, decisions to seek care outside the home were often collaborative. A husband’s influence on decision-making could cause significant delays, but only two mothers stated that their husbands actively prevented them from seeking care outside the home.

For approximately one-third of mothers interviewed, husbands were characterized as passively involved in the decision to seek care outside the household, especially in West Hararghe. Several stated that when their husbands were unavailable, they would access care or social support without them. Many husbands suggested that they deferred decision making of initial care-seeking choices to their wives, elder family members and sisters, stating this is a *“woman’s concern*.” Sometimes, members of a mother’s lay referral network were enlisted to support a mother’s desire to seek care outside the household and persuade hesitant husbands.

### Delay in seeking care outside the home

Most mothers seeking care outside the household stated they waited for more than 24 h before leaving the home to seek care for their sick child. The most common reasons given for delaying care-seeking outside the home related to negotiations and disagreements within the household and desired care-seeking choices. Additional reasons given were: uncertainty about illness progression, difficulties with access to treatment, apprehensions about presenting to providers and the exigencies of daily life.

In nearly half of households there was some disagreement on the type and timing of care-seeking. Disagreements related to expectations of costs associated with care-seeking outside the home, differing illness interpretations and, related to this, the perceived likelihood of recovery following a particular course of care-seeking. The majority of disagreements were between husbands and wives, often relating to expectations of cost, but were also between a wife and her husband’s elderly relatives, relating to differing illness interpretations. The dynamics and level of disagreement and length of delays varied considerably as portrayed in the following quote:

*I told my husband we must not wait because her breathing was worsening. He worried over money and did not want to give money for the wrong treatments. We argued over the right actions. After two or three days, I received assistance from neighbors who said they would lend money and talk to my husband. He then went to buy medicines.* (IDI, mother, 25–29 years old, agriculture, Jimma)

This account also suggests that some mothers are able to actively utilize social support and care providers to resolve disagreements and validate care-seeking actions.

Uncertainty in recognizing a child’s symptoms and illness continued as caregivers monitored and assessed illness progression in the home, leading to potential delays in care-seeking outside the home. Despite suggesting that their child’s illness was serious, many caregivers still expressed uncertainty regarding particular, often unfamiliar, symptoms and the transition from common to severe illness, weighing these against the social and economic implications of care-seeking actions. Many continued to delay in the hope that their child’s illness was self-limiting and had difficulties in determining meanings associated with changes in symptoms and the appropriateness of care-seeking actions.

Another common source of delayed care-seeking outside the home related to availability and access to treatment. This could be due to both a caregiver’s anticipation of difficulties or actual experience. Some caregivers noted apprehension about the economic effects on the household and the difficulties of limited access to financial resources of households. The cost and efforts of transporting a child were a primary concern, especially during the wet season and for female caregivers, and frequently led to delays as caregivers organized resources and transportation.

Nearly all caregivers agreed that care taking of a child, especially during sickness, is primarily a mother’s responsibility. However, many informants stressed that this is only one of the many responsibilities of a woman in rural Oromia, responsibilities that may compete with a mother’s ability to recognize and act upon a child’s illness. One mother ended an FGD with this a statement:

*We are proud to be mothers and do our duties to watch over our children, but we women are overworked. We have many responsibilities in our household and in our village. When our child is sick, we must leave our other children unsupervised and unfed. We may spend money if it is serious, but we have no money. Our lives are difficult and our children suffer.* (FGD, mothers, West Hararghe)

### Perceptions and choice of care provider

Care-seeking behaviors reported in this study revealed a broad range of providers reflecting a mixed market of, often overlapping, public, private, biomedical and traditional health services. The most common options at the local community level mentioned by caregivers were: drug peddlers, informal traditional herbalists, rural drug vendors and HEWs at the health post. A small number of caregivers also mentioned public health centers and private clinics and pharmacies in the nearest urban center. Other less common sources mentioned included traditional healers and spiritualists.

For caregivers in this study, perceptions of appropriateness and efficacy of a provider or treatment, both of which acted to shape a caregiver’s preferences, influenced the decision to utilize a particular course of care. These preferences were weighed against perceptions and realities of accessibility of care sources. Both preferences and accessibility were moderated by the inclusion of a caregiver’s lay referral network.

The “power” (*dawa jaba*) and efficacy of treatments and drugs distributed by a provider was the primary consideration for most caregivers in preferring one source to another. In general, local herbs were seen as the least powerful treatment and biomedical drugs the most powerful. Perceptions of the power of *qoricha Oromo*, distributed by elders, herbalists and traditional healers varied depending on attitudes toward traditional medicine. For those with positive attitudes, the power of *qoricha Oromo* was largely based on its ability to counter potential supernatural causes for a child’s condition and to cure *lafu*. For biomedical treatments, the form rather than nature indicated the power of a drug for most participants. ORS and tablets were seen as having the least power followed by syrups and finally, injections, perceived as the most powerful. The source of drugs also contributed to the power of a treatment with urban sources perceived as superior to community-based services. A small number of informants suggested that perceptions of the ability and willingness of a provider to dispense a biomedical drug of desired power were influential in treatment choice. These caregivers suggested that urban providers and rural drug vendors were often the most able to provide powerful drugs, drug peddlers were the most willing and HEWs were the least willing and able.

Despite these perceptions, many caregivers suggested that the most powerful drug was not always the most effective and that they chose a treatment of appropriate power based on a caregiver’s provisional understanding of their child’s illness.

*P*_*1*_ *: A child’s blood is weak.*

*P*_*2*_ *: Some children are lafu.*

*P*_*1*_ *: Yes, these children are very weak. We do not give these children powerful drugs because they may die. These are only for adults and older children.*

*P*_*3*_ *: A child can take powerful drugs if they are prepared.*

*I: How do you prepare a child?*

*P*_*3*_ *: A child must be given strength. There are herbs that elders know to give strength to a child. They know herbs to protect a child against harm.*

*P*_*1*_ *: And to strengthen medicines.* (FGD, mothers, West Hararghe)

Like these participants, several caregivers expressed concern about the potential side effects of powerful drugs. This perception was especially pronounced for very young children (under the age of 2 months) and many caregivers deemed biomedical drugs harmful for these children. For these reasons, illnesses of lower perceived severity and very young children were often treated with herbal medicines instead. As related by these participants, herbal medicines are useful for strengthening children, mitigating the side effects of powerful drugs and amplifying the power of other treatments. In the many cases in which uncertainty about the severity and nature of a child’s illness persisted, caregivers tended to engage in a trial-and-error search aimed both at minimizing uncertainty and alleviating a child’s symptoms. These strategies typically began with sources such as herbalists that are perceived to provide greater understandings of a child’s illness and to have less potential for dangerous consequences such as using powerful drugs.

In addition to ideas about efficacy in relation to an illness interpretation, provider preferences also commonly depended upon expectations of caregiver-provider interactions. When discussing her experiences interacting with health centers and HEWs one mother stated:

*I am proud to be a good Oromo mother with some education, but I am not treated well when I go to government services for my child. They see us as superstitious and ignorant. They ask us why we did not come sooner and tell us we are to blame if our child dies.* (FGD, mothers, West Hararghe)

Like this mother, some caregivers noted instances in which they felt disrespected and judged as “ignorant” or blamed as “bad mothers.” The expectation of being negatively judged was commonly reported as an explanation for avoiding or delaying care-seeking from government health centers and, at some sites, HEWs and opting for other providers where they would receive more respectful care. Most caregivers noted expectations of understanding more about their child’s condition such as the cause and illness label during a provider interaction such as the following exchange:

*R: We did not know the cause of this sound [“grunting”]. We thought she suffered from qora [flu] but this was not qora. We asked the village herbalist to explain the meaning of her [child]. We did not know if she had medicines for this, but she [herbalist] told us that it was serious and was because her lungs were fighting the cold wind. We went to the health post immediately after.*

*I: Why did you not immediately go to the health post?*

*R: The worker will not tell us why our child suffers. I don’t understand her [HEW] words.* (IDI, mother, 30–34 years old, agriculture, Jimma)

For this reason, elders, herbalists, and to a lesser extent, HEWs, were often preferred for their willingness to provide culturally and socially understood meanings of their child’s illness. This understanding was instrumental in facilitating respectful communication between a caregiver and provider and led many mothers initially uncertain of their child’s illness to prefer these providers. Despite these preferences, most caregivers noted a range of access-related barriers preventing them from acting upon their desired care-seeking course. Although urban care options such as health centers, private clinics and pharmacies were commonly perceived as the source of the highest quality of service and treatments, these sources were infrequently utilized due to significant financial and transportation barriers. Two study sites were within a two-hour walk to an urban center and caregivers from these sites accounted for all utilization of private urban providers and by passing of the health post to the health center. For the remaining sites, except for instances of referral to a health center, care-seeking was predominantly from community-based providers. While HEWs and health posts were designed to bring free and quality services closer to rural households, caregivers (especially from households greater than one-hour walk) still noted long distances, geographical barriers such as hills, forests and rivers, poor paths, inadequate transportation services and difficulties carrying sick children—all compounded during the wet season—as significant barriers to accessing the health post as noted by the following informant:

*We are told to visit the health post when our child is sick. Drugs are free and that is good for poor people like us. But it is difficult to carry a sick child from my household. The health post is in [sub-village] which is very far. During rains, I must hire transportation but there are no motorbikes, no bijaj [motorized taxis] to take us. If I carry him [child] I may injure him more.* (IDI, mother, 25–29 years old, agriculture, Jimma)

Caregivers noted that HEWs were often unavailable, especially during nights and weekends when urgent treatment might be needed. Rural drug vendors, while often perceived as distant and potentially costly, were often seen as more accessible because mothers could request that their husbands travel and did not require the presence of the child. In contrast to these sources, most caregivers mentioned that relatively inexpensive treatments could be secured from herbalists and drug peddlers (at general stores and markets) and were relatively easy and convenient to access.

Many caregivers suggested that they formed these perceptions based on previous child illness experiences. However, the majority also suggested that their lay referral networks were heavily influential in forming and modifying these perceptions. Similar to decisions in preceding stages of care-seeking, the decision on where to seek care was a socially negotiated process. A caregiver’s lay referral network was often instrumental in providing awareness of different provider options, giving treatment advice and supporting caregivers to overcome access barriers to desired sources of care such as by lending money and assisting with the transport of children.

On the other hand, some mothers noted contested care and treatment strategies between their own treatment preferences and that of influential family or community members. These mothers often felt pressured into acting first on the preferences of family and community members rather than their own desired care-seeking strategies. Caregivers suggested that elderly family members tended to prefer first trying herbal medicines and *qoricha Oromo* before utilizing other sources. Father informants frequently saw rural drug vendors and, to a lesser extent, drug peddlers as most effective in treating their children. In other instances, mothers were actively prevented or felt disempowered from acting on her preferences.

*I agreed with my neighbor woman that she [child] needed to go to the health post. I told my husband we must take her to the health post immediately because her fever was increasing. He said to wait and told me to get drugs from the market the next day. I did not want to act against his words but took her to my husband’s family. They agreed that I should go immediately to the health post.* (IDI, mother, 30–34 years old, agriculture, West Hararghe)

As this mother’s experience demonstrates, a small number of mothers were able to reassert their preferences by appealing to more senior members of their lay referral networks.

### Use of multiple providers and provider switching

Nearly all caregivers in this study suggested the use of multiple providers over the course of their child’s illness. The most common pathways mentioned in IDIs are given in Table 3. Self-treatment, particularly with the use of herbs and assistance of elders, was the most common first step of care and treatment. Unlicensed drug vendors were another form of self-treatment commonly utilized early in the care-seeking process. Use of these sources was regarded as successfully resolving a child illness in more than one-third of illness episodes. For unresolved illnesses, caregivers were increasingly likely to transition to biomedical options such as rural drug vendors and HEWs at the health post as illness and care-seeking progressed. There were small differences by illness type with caregivers discussing fever or respiratory ailment being more likely to be addressed using drug peddlers, rural drug vendors or traditional healers. Caregivers of children with diarrhea were more likely to engage in herbal and *qoricha Oromo* treatments. Finally, those illnesses labeled as *ibidda*/*cimma* were more likely to lead to the engagement of elders as well as HEWs at the health post.

**Table 3:** Care-seeking pathways followed by two or more caregivers (presented in order of frequency)

1.	Home treatment → Traditional healer
2.	Home treatment only
3.	Home treatment → Informal drug vendor
4.	Home treatment → Traditional healer → Informal drug vendor
5.	Home treatment → Traditional healer → Health post
5t.	Home treatment → Health post
7.	Home treatment → Traditional healer → Health post → Traditional healer
7t.	Home treatment → Health post → Health center
9.	Traditional healer → Informal drug vendor → Health center
9t.	Traditional healer → Health center → Traditional healer
9t.	HEW only

While a return of *jolii si’a ina qaba* was the primary goal of treatment, a number of secondary objectives of treatments were expressed. Some care sources, especially those used in early care-seeking steps, were also utilized in order to learn more about the illness (e.g. illness label and severity) as caregivers gauged changes in the child’s condition in response to treatments. Herbal treatments were common for this purpose. Some treatments, such as drugs obtained at village shops and marketplaces, were used for limited purposes, as in the case of one particular symptom, rather than aimed at the illness as a whole. Still other treatment sources were used to complement a concurrent treatment such as the use of both traditional herbs and biomedical drugs whereby the former “weakens” the illness or increases the power of the latter. Finally, some treatments, particularly spiritual and traditional healers consulted in later care-seeking steps, were used to cure or prevent *lafu*. Evaluations based on both primary and secondary expectations were instrumental in a caregiver’s decision to terminate care-seeking, continue with the current course of action or switch care providers.

When a treatment course failed to meet caregiver expectations, the majority perceived this outcome as due to the lack of fit between the child’s illness and the medicine’s power and appropriateness.

*Herbs were useless for him [child]. They were too weak but I tried because I could gather them quickly. His breathing was with a’adu [grunting] and needed stronger medicines. I said he should have an injection for this problem and went to the health post* (IDI, mother, 18–24 years old, agriculture, Jimma)*.*

Additional reasons given for transitioning to alternate care sources included wanting to maintain the appearance of being a “good mother”, as women did not want to be perceived as “taking incorrect actions,” if the treatment resulted in their child’s condition worsening. As one woman who described seeking multiple sources described “*I did not want to be called careless. I tried everything a mother here can do to here to stop the diarrhea*”. Other reasons discussed included a caregiver’s dissatisfaction with personal treatment by a provider, appeasement of influential members of a lay referral network, a change in the ability to access a provider or treatment and/or a change in the child’s condition or interpretation of a child’s illness.

## Discussion

We investigated the context of care-seeking for child illness and especially, low rates of use of “appropriate” providers and treatments (e.g. regulated sources of drugs and evidence-based medications) for sick children. With recent investments in primary care, after 10 years of the HEP and 2 years of scale up of iCCM, still only 10% of sick children are taken to HEWs at health posts for treatment [17]. Our findings suggest that low utilization of this program is not explained by low awareness or even low perceived need of this healthcare option, but is linked to sociocultural factors such as how caregivers understand and perceive an episode of child illness and how it is negotiated within families and communities.

Similar to a study conducted by Alvesson et al. in Laos, our findings suggest that deconstructing uncertainty in the development of care-seeking behaviors is critical to understanding strategies used in a context with multiple healthcare options [22]. Uncertainty was a common theme expressed by caregivers throughout the care-seeking process for a sick child, relating to finding the correct illness label, identifying the cause(s) of an illness, selecting the right provider and treatment course, and mobilizing household resources. The contested nature of illness identification suggests that social norms act as only a partial guide to care-seeking behaviors and individual actions can play a considerable role. The distinctions between traditional (*wabiya*) and modern (*tijanaya*) illnesses, highlighted in interviews with HEWs and VCHWs and even within FGDs as normatively guiding treatment choice, were often ambiguously portrayed in individual caregiver narratives. In IDIs, caregivers more often discussed illness labels and understandings in terms of those that were familiar/non-familiar illnesses or those that required “powerful/weak medicines.” Leach et al. similarly argue, based on work done in Guinea, that distinctions between modern/traditional or public/private care options are more often invoked by health professionals and that new “therapeutic landscapes” are necessary to understand health-seeking behaviors in sub-Saharan Africa [14]. Caregivers minimized uncertainty over the course of a child’s illness by: “giving a name” to an illness in order to render it understandable in terms of a particular care-seeking course and to distinguish the illness severity; enlisting both “laypeople” and “experts” (including hybrid providers such as elders and local shop vendors) to provide social support to decrease uncertainty and enable care-seeking.

A more salient distinction described was that between those actions that constitute a “good” versus “bad” mother in terms of making socially and culturally acceptable decisions in the care-seeking process for a sick child. Care-seeking decisions may reflect the social significance of being a responsible caregiver and showing respect for household and cultural norms as suggested by Alvesson et al. based on work in Laos [22]. A consistent theme across care-seeking decisions and processes was the potential for blame for a child’s illness, worsening condition or the failure of treatments lending a moral dimension to care-seeking strategies. Some mothers suggested taking actions to avoid this label by choosing providers (e.g., biomedical) to be recognized as *tijanaya* or providers (e.g., *qoricha Oromo*) that more positively affect their sense of self-worth, trust and community. The moral dimensions of caregiving are often overlooked in the literature, but have been outlined by Ricketts and Goldsmith, giving moral dimensions a central place in their care-seeking framework [23].

According to Thiede, trust assumes a key position within the transactional process of information exchange, and use of different care providers [24]. Despite the official policy of selecting HEWs from the local community, this was not always achieved in practice. The requirement of literacy and a relatively high degree of education often meant that an HEW was from a relatively better-off socio-economic strata compared to most informants in this study. This relatively asymmetrical relation in terms of social status often led to mistrust of HEWs by caregivers, who felt marginalized from the services of HEWs at the health post. The lack of cultural sensitivity and instances or rumors of HEWs viewing caregivers as “ignorant” often increased the likelihood of choosing alternative sources. As well, the government of Ethiopia is largely controlled by members of the Tigrayan ethnic group despite the Oromo people constituting a majority of the Ethiopian population. Recent protests by Oromo students and communities have led to violent clashes with government police and military forces [25]. Consequently, caregivers in some FGDs suggested a low-level of trust in government-run health programs which one participant described as a way to, “*control the Oromo people*.” In contrast, informal providers such as elders practicing *qoricha Oromo* and local shop owners were trusted members of a caregiver’s community. Moreover, these providers relied on traditions of the Oromo and were described as “*one of us.*”

A number of studies have demonstrated, similar to our findings, that illness interpretations and care-giving strategies are a deeply social affair and often developed through ongoing consultation within a caregiver’s lay referral network [10, 12, 26, 27]. A mother’s husband, extended family, and neighbors were influential in her lay referral network that also assisted in minimizing uncertainties associated with childhood illness and care-seeking. Caregivers in this study saw members of their social network as valuable sources of social and financial support and practical advice, but several participants pointed out that this could lead to considerable delays in seeking care and especially from formal sources of care. For some mothers, autonomy in care-seeking decisions and the potential for blame increased as a child’s illness progressed and additional social actors were engaged. However, many mothers suggested the ability to re-assert her preferences by selectively engaging social actors and “experts” within a lay referral network to take desired actions. We found that the large majority of mothers were open to and even preferred the use of HEWs at the health post.

In contrast to other sub-Saharan African settings such as outlined by Tolhurst et al. in Ghana and Kamat in Tanzania [28, 29], the influence of gender of the caregiver and access to power on household decision-making on care-seeking for child illness appeared to be less restrictive for our sample of informants. Most mothers suggested a more collaborative nature or in the least, a passive role for husbands, especially during early stages care-seeking. Although a husband’s role tended to increase as a child’s illness became more severe, required care-seeking outside the home and financial resources, most participants described a collaborative relationship with relatively few instances of husband’s withholding resources or prohibiting care-seeking outside the home.

Another strong theme related to the nature of drugs and treatments received from particular sources. Many studies have found that biomedical drugs, and particularly injections, are perceived as the most powerful form of medicine [13, 30, 31]. However, this study adds nuance to these findings, indicating that it does not necessarily follow that they are most appropriate, while herbal medicines also had gradients of power and appropriateness.

Although this was a qualitative study with a limited sample size, the high rates of use of informal providers including informal drug vendors and elders practicing *qoricha Oromo* and of using multiple providers challenge findings from quantitative surveys suggesting low use of informal sources and low rates of care-seeking from multiple sources in Ethiopia [17]. Our findings give some support to the hypothesis that caregiver beliefs about childhood illness and preferences for alternative sources of care are important reasons for low use of formal services such as discovered by Mebratie et al. conducting a qualitative study among a similar population in Ethiopia [32]. The majority of caregiver care-seeking strategies in this study involved use of informal sources of care, similar to other sub-Sahara African settings discussed by Colvin and colleagues’ review [13]. It is likely that stigmatization associated with these sources and difficulties in understanding response categories could potentially lead to underreporting of the use of these services in our study as cautioned by Ansah and Powell-Jackson [33]. For example, there was widespread use of elders and *qoricha Oromo*, which was not always perceived as “traditional medicine.” Consequently, the large role these individuals and treatments, as well as informal drug vendors, played for caregivers in this study are potentially being underappreciated. Similar observations have been made in other research regarding elder/providers and informal vendors in Ethiopia [29]. We found that most caregivers left the household to seek care, often promptly after recognizing symptoms. However, there was a cultural taboo observed in most research sites whereby caregivers of very young children did not leave the home for treatment. This has serious implications for newborn care and has been documented in Ethiopia [34].

This study is subject to a number of potential limitations. Misinterpretation and mistranslation were potential risks for our data. We sought to minimize these risks through the use of data collectors whose first language was Afan Oromo and had strong English speaking skills and through the use of careful back-translation of transcripts. Second, it is possible that interviewees expressed what they perceived to be appropriate or socially desirable responses. We sought to minimize this risk by conducting semi-informal, private interviews with informants and by asking questions in multiple ways to allow responses to be triangulated. Participants were purposively selected with assistance from local leaders and health post staff and therefore there is the potential that the participants were not representative of the community as a whole. Although our sample size was relatively small, it did result in saturation of findings, potentially reducing the impact of non-probability sampling. This study was restricted to Jimma and West Hararghe zones of Oromia region. All caregivers involved in this study were Afan Oromo-speaking members of the Oromo ethnicity. Findings from this study may not be generalizable to other Ethiopian regions or ethnicities. Nevertheless, in relating the findings to other published literature, we have often found convergence with other settings and populations.

## Conclusions

This paper contributes to the growing body of qualitative research by following the care-giving experiences of rural Ethiopian caregivers from the act of recognizing a child’s illness to household decision-making processes to evaluations of provider and treatment choices. Our research suggests that it is essential for existing and future interventions to recognize the dynamic nature of the entire course of care-seeking in this setting. This includes acknowledging the uncertainty inherent in recognition and response to childhood illness, the social nature of care-seeking, the burdens and challenges faced by mothers and the continuing popularity and often complementary nature of alternative, community-based sources of care.

## Additional file

Additional file 1: Research instruments for “A Qualitative Exploration of Care-Seeking Pathways for Sick Children in the Rural Oromia Region of Ethiopia”. This file contains the general focus group discussion guide for caregivers, in-depth interview guide for caregivers and in-depth interview guide for health workers used for eliciting data for the manuscript. (DOCX 38 kb)

## Abbreviations

FGD: Focus group discussion
HEP: Health extension program
HEW: Health extension worker
iCCM: Integrated community case management
IDI: In-depth interview
MDG: Millennium Development Goal
ORS: Oral rehydration solution
VCHW: Voluntary community health worker
