# Supplementary material for: A qualitative exploration of care-seeking pathways for sick children in the rural Oromia region of Ethiopia
Source: BMC Health Serv Res. 2017 Mar 9;17:184. doi: 10.1186/s12913-017-2123-5 (PMC5343381; doi:10.1186/s12913-017-2123-5)
Supplement: Additional file 1: — Research instruments for “A Qualitative Exploration of Care-Seeking Pathways for Sick Children in the Rural Oromia Region of Ethiopia”. This file contains the general focus group discussion guide for caregivers, in-depth interview guide for caregivers and in-depth interview guide for health workers used for eliciting data for the manuscript. (DOCX 38 kb) [file 12913_2017_2123_MOESM1_ESM.docx]

**Additional files legend**

*File name:* Questionnaire_Careseeking Ethiopia_Feb 24 2017.docx

*Title:* Research instruments for “A Qualitative Exploration of Care-Seeking Pathways for Sick Children in the Rural Oromia Region of Ethiopia”

*Description:* This file contains the general focus group discussion guide for caregivers, in-depth interview guide for caregivers and in-depth interview guide for health workers used for eliciting data for the manuscript.

**Focus group discussion guide for caregivers**

***Module 1***

Q1

What are the main child health problems in your community?

Do many young children (under 5) die in this area?

What do they die from?

Q2

What causes malaria and what are the symptoms?

If a child has malaria, what should a mother do?

What causes diarrhea and what are the symptoms?

If a child has diarrhea, what should a mother do?

What causes pneumonia and what are the symptoms?

If a child has pneumonia, what should a mother do?

***Module 2***

Q3

For child illness, do you use traditional medicine/home remedies? What for?

Does your family use traditional medicine/spiritual healer for child illness?

What other sources are used for child illness? (Ask whether use, what for)

Q4

What kind of help do husbands/family provide when a child is ill?

Who else provides help when a child is ill? What do they do?

Q5

What cultural beliefs influence child illness and treatment seeking in your community?

Q6

Where do you get information about child illness?

What measures should be taken to improve the community’s knowledge about child illness?

***Module 3***

Q7

What are the main challenges your family faces in going to the health post?

What are the reasons that some families do not take their child for treatment at the health post?

Does cost or distance make accessing the health post difficult?

Q8

What are the solutions to these challenges/barriers?

What can be done to improve the health of children in this area?

**In-depth interview guide for caregivers**

***Module 1***

Q1

What are the main child health problems in your community?

Do many young children (under 5) die in this area?

What do they die from?

Q2

When did you first notice that your child was sick?

What caused your child’s illness?

What is the name of your child’s illness?

What are the symptoms of your child’s illness?

***Module 2***

Q3

When your child was sick with X, what actions did you take?

(prompt a narrative focusing on what was done, who child was taken to, who was involved in decisions, where child was taken, the amount of time elapsed between steps and why each step was taken)

Q4

When a child is ill, who do you tell?

What kind of help did your husband/family provide when your child is ill?

Who takes the decision to treat the child?

Who else provided help? What do they do?

Q5

Where do you get information about child illness?

***Module 3***

Q6

Depending on whether used health post, why or why not used?

What are the main challenges your family faces in going to the health post?

Did cost or distance make accessing the health post difficult?

Q7

What are the solutions to these challenges/barriers?

What can be done to improve the health of children in this area?

**In-depth interviews for health workers**

***Module 1***

Q1

What are the main child health problems in your community?

Do many young children (under 5) die in this area?

What do they die from?

***Module 2 omitted***

***Module 3***

Q2

In your opinion, why do caregivers take their child to the health post in this community?

In your opinion, why do caregivers not take their child to the health post in this community?

Where do caregivers take their children to get care and treatment for a sick child in this community?

Q3

Are there particular populations that do not use the health post? Why?

What kind of help did your husband/family provide when your child is ill?

Who takes the decision to treat the child?

Who else provided help? What do they do?

Q4

What are the solutions to these challenges/barriers?

What can be done to improve the health of children in this area?
